# Nanoparticle‐Mediated Cuproptosis and Photodynamic Synergistic Strategy: A Novel Horizon for Cancer Therapy

**DOI:** 10.1002/cam4.70599

**Published:** 2025-01-27

**Authors:** Junrui Zhang, Anren Zhang, Yibing Guo, Guoliang Miao, Shengchang Liang, Jie Wang, Junhong Wang

**Affiliations:** ^1^ Gansu University of Chinese Medicine Lanzhou Gansu Province China; ^2^ Gansu Provincial Hospital Lanzhou China; ^3^ School of Basic Medical Sciences Lanzhou University Lanzhou Gansu Province China; ^4^ Department of General Surgery The First People's Hospital of Baiyin (Third Affiliated Hospital of Gansu University of Traditional Chinese Medicine) Baiyin China

**Keywords:** cancer, cuproptosis, PDT, therapy nanomaterials

## Abstract

**Background:**

Photodynamic therapy (PDT) is a noninvasive cancer treatment that works by using light to stimulate the production of excessive cytotoxic reactive oxygen species (ROS), which effectively eliminates tumor cells. However, the therapeutic effects of PDT are often limited by tumor hypoxia, which prevents effective tumor cell elimination. The oxygen (O_2_) consumption during PDT can further exacerbate hypoxia, leading to post‐treatment adverse events.

**Objectives:**

This review aims to explore the potential of cuproptosis, a recently discovered copper‐dependent form of programmed cell death, to enhance the anticancer effects of PDT. Cuproptosis is highly dependent on mitochondrial respiration, specifically the tricarboxylic acid (TCA) cycle, and can increase O_2_ and ROS levels or decrease glutathione (GSH) levels, thereby improving PDT outcomes.

**Methods:**

The review discusses the latest research advancements in the field, detailing the mechanisms that regulate cuproptosis and PDT. It also explores how nanoparticle (NP)‐based strategies can be used to exploit the synergistic potential between cuproptosis and PDT. The article examines the prospects of synergistic anticancer activity guided by nanodelivery systems, which could overcome the challenges associated with hypoxia in cancer treatment.

**Conclusions:**

The combination of cuproptosis and PDT, facilitated by NP‐based delivery systems, presents a promising approach to enhance the effectiveness of cancer therapy. The review concludes by discussing the challenges and future research directions for this combination therapy, highlighting the need for further investigation into the mechanisms and optimization of treatment strategies to improve outcomes in cancer treatment.

## 1 Introduction

Cancer is a major public health issue and a leading cause of disease burden worldwide. According to the latest statistics from the International Agency for Research on Cancer, there were nearly 20 million new cancer cases in 2022, with approximately 9.7 million deaths from cancer [1]. Therefore, advancing cancer research by developing innovative therapies and treatments that precisely target and effectively manage this disease is crucial. Traditional methods of treating cancer include surgery [2], radiotherapy [3], and chemotherapy [4]; however, issues such as trauma, side effects, and drug resistance hinder their effectiveness. Recently, several novel therapeutic modalities have been developed, including chemodynamic therapy [5], photodynamic therapy (PDT) [6], sonodynamic therapy [7], photothermal therapy [8], and immunotherapy. These approaches are currently in preclinical stages and require further development and refinement.

Copper (Cu) is a vital trace metal that plays essential roles in numerous physiological processes, including mitochondrial respiration, antioxidant mechanisms, iron absorption, and detoxification pathways [9, 10]. Cu can induce cell death through various mechanisms such as triggering oxidative stress, inhibiting proteasomes, and the recently discovered pathway called “cuproptosis.” In 2022, Tsvetkov et al. revealed a novel Cu‐induced cell death pathway that was distinct from apoptosis, pyroptosis, necroptosis, and ferroptosis [11]. Cuproptosis is initiated by the direct binding of Cu ions to the sulfated components of the tricarboxylic acid (TCA) cycle within the mitochondrial respiratory chain. This interaction leads to the aggregation of sulfated proteins, resulting in diminished activity of Fe–S cluster proteins and subsequent induction of protein toxic stress, ultimately culminating in cellular demise. Tumor cells exhibit elevated metabolic activity and energy expenditure, which are intricately linked to mitochondrial function, resulting in Cu‐dependent cell death associated with the TCA cycle. This presents novel opportunities for targeted eradication of tumor cells.

PDT, a light‐driven therapeutic modality, has garnered significant attention owing to its noninvasive photochemical reaction that elicits localized cell apoptosis. Although some cutting‐edge methods related to PDT are still in the preclinical research phase and require further development and optimization, PDT, as a clinically validated cancer treatment modality, has been widely adopted as a minimally invasive treatment strategy. Compared to traditional surgical procedures, PDT holds a significant position in clinical practice because of its lower invasiveness, fewer side effects, and shorter treatment duration [12, 13]. PDT is contingent on the local or systemic administration of photosensitizers (PSs), which selectively accumulate in pathological tissues through mechanisms such as the enhanced permeability and retention effect, targeting ligands, and microenvironmental responses [14]. Upon exposure to specific wavelengths of light, these PSs generate cytotoxic reactive oxygen species (ROS), leading to photochemical and photophysical reactions that destroy cancer cells [6, 15, 16, 17]. Furthermore, literature indicates that PDT can disrupt the vascular structure of tumors, depriving cancer cells of oxygen (O_2_) and vital nutrients [18]. PDT effectively triggers tumor cell elimination, causing the release of tumor‐associated antigens (TAAs) that stimulate an immune response. Simultaneously, it enhances the activation of immune cells such as dendritic cells and T lymphocytes, thereby augmenting the antitumor capabilities of the immune system [19]. These salient attributes render PDT an extremely promising therapeutic approach for cancer.

However, cuproptosis and PDT have certain limitations. The delivery of Cu ions in the form of Cu^+^ to tumor cells contributes to cuproptosis [20]. Unfortunately, because of the low redox potential of Cu^2+^/Cu^+^ (≈0.16 V), Cu^+^ is extremely unstable and readily oxidizes to Cu^2+^ [21]. Moreover, the Cu efflux mechanism and intracellular reducing substances with high expression levels could effectively attenuate the cuproptosis effect. Overexpressed glutathione (GSH) in the tumor microenvironment (TME) can act as a Cu‐chelating agent, removing excess Cu ions and safeguarding tumor cells against cuproptosis [22]. Simultaneously, elevated levels of the antioxidant GSH impede ROS production, significantly affecting PDT efficacy [23]. Additionally, high O_2_ consumption during PDT exacerbates TME hypoxia, whereas Cu‐based nanomaterials generate ROS and O_2_ via Fenton‐like reactions to enhance the effectiveness of PDT and alleviate tumor hypoxia [24].

Given that PDT can effectively synergize with cuproptosis, an increasing number of researchers are focusing on their interplay in cancer treatment. Recently, there has been a surge in studies exploring their combined applications, yielding promising advancements. Although numerous reviews have addressed individual investigations of cuproptosis and PDT, there remains a paucity of studies examining their synergistic anticancer effects. This study elucidates the antitumor mechanisms underlying cuproptosis and PDT, discusses their respective advantages and limitations, and reviews emerging nanoparticles (NPs) designed for the synergistic treatment of cancer through the combination of these two modalities, alongside other therapeutic approaches. Finally, we highlight the current challenges and future directions of synergistic cancer therapies involving cuproptosis and PDT.

## 2 The Mechanisms of Cuproptosis and PDT for Cancer Therapy

### 2.1 The Mechanisms of Cuproptosis

**FIGURE 1:**
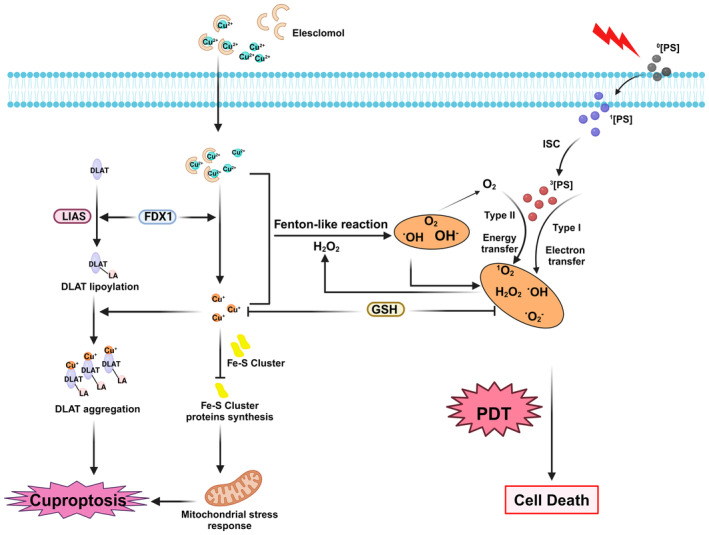
Schematic mechanism of synergistic cancer therapy of cuproptosis and PDT. Type I and Type II reactions occur when a ^0^PS absorbs a photon and becomes excited to the ^1^PS. The excited PS can undergo ISC to form a triplet‐state ^3^PS. Type I involves electron transfer; Type II involves direct energy transfer. The Cu^2+^ released by elesclomol (ES) is reduced to Cu^+^ by the mitochondrial enzyme FDX1, leading to oligomerization of fatty acylated DLAT and instability of the Fe–S cluster protein, ultimately resulting in cuproptosis. GSH can act as a Cu‐chelating agent to remove excess Cu ions and as a reducing agent to remove ROS, significantly affecting the efficacy of cuproptosis and PDT. Cu ions can produce ROS and O_2_ through a Fenton‐like reaction. ^0^PS, singlet ground state photosensitizer; ^1^PS, singlet excited state photosensitizer; ^3^PS, excited triplet state photosensitizer; DLAT, dihydrolipoamide S‐acetyltransferase; FDX1, ferredoxin 1; GSH, glutathione; ISC, intersystem crossing; LA‐DLAT, lipoylated DLAT; LIAS, lipoyl synthase; ROS, reactive O_2_ species. Created with BioRender.com.

Despite decades of research into Cu‐induced cell death, it was not until 2022 that Tsvetkov and colleagues introduced the concept of “cuproptosis” [25]. This regulatory mechanism of cell death is primarily associated with the lipoic acid pathway and mitochondrial respiration [26]. Tsvetkov et al. found that ES, a Cu ionic carrier, can form a complex with Cu^2+^ and the ES‐Cu^2+^ complex is transported to the mitochondria. Excessive binding of Cu^2+^ to lipoylated dihydrolipoamide S‐acetyltransferase (DLAT) leads to abnormal polymerization of DLAT and an increase in insoluble DLAT, inducing cell death. Ferredoxin 1 (FDX1), which serves as an upstream regulatory factor in protein thioacylation, regulates the thioacylation of DLAT. Moreover, FDX1 reduces Cu^2+^ to a more toxic form of Cu^+^, resulting in diminished stability and the synthesis of Fe–S cluster proteins [27], ultimately triggering mitochondrial stress responses and causing cell death (Figure 1).

### 2.2 The Mechanisms of PDT

The principle of PDT is that specific light can excite PSs, which then transfer energy to the surrounding O_2_ molecules, generating ^1^O_2_ and ROS, causing irreversible and permanent death of cancer cells [28]. PDT typically consists of three essential components: PSs, excitation light, and molecular O_2_ [29]. Although these components are not toxic individually, their combination triggers a photochemical reaction that produces cytotoxic ROS. The photochemical PDT process is illustrated in Figure 1. Upon exposure to the light of a specific wavelength, PS absorbs photons and becomes excited, transitioning from the singlet ground state (S0) to the singlet excited state (S1), and subsequently undergoes a transition to the triplet excited state (T1) [30]. Following this, photochemical reactions occur to generate ROS [31]. This process generates ROS via two types of photochemical reactions. Upon exposure to specific wavelengths of light, PSs can be excited and react to endogenous O_2_ to produce radicals such as superoxide radicals (•O_2_^−^) and hydroxyl radicals (•OH) in Type I reactions [15, 32, 33]. In contrast, Type II reactions involve the direct energy transfer from the excited PS to O_2_, resulting in the formation of highly reactive singlet oxygen (^1^O_2_), which is significantly cytotoxic [34, 35, 36]. It is important to note that although Type I reactions can proceed in the presence of O_2_, they are not strictly O_2_‐dependent [37]. On the other hand, Type II reactions are markedly O_2_‐dependent, as the production of ^1^O_2_ is contingent upon the energy transfer to O_2_ [38]. PDT inhibits tumor growth through three mechanisms: firstly, by using ROS to directly kill the tumor [39]; secondly, by targeting tumor blood vessels [40]; and thirdly, by activating the immune system to induce inflammation and immune responses against tumor cells [41].

### 2.3 The Relationship Between the Mechanisms of Cuproptosis and PDT

A previous study has demonstrated that Cu‐containing catalytic reactions, namely, Fenton‐like reactions, are more favorable in the TME than those involving iron (Fe) and manganese (Mn) [21]. Equations (1) and (2) depict the Cu‐based catalytic reactions. The catalytic efficiency of Cu^+^ in Fenton‐like reactions was remarkably high under slightly acidic and neutral conditions. The generation of •OH by this reaction triggers a cascade of free radical reactions, leading to oxidative modification of phospholipids within cellular membranes, rupture of plasma membranes, and ultimately resulting in cell death [42]. The utilization of Cu‐based nanosubstrates has the potential to synergistically enhance the effects of PSs, thereby maximizing their combined efficacy. Type I cytotoxicity induced by PDT occurs through electron transfer to generate ROS and H_2_O_2_, whereas Type II cytotoxicity occurs when PS interacts with O_2_ to generate ^1^O_2_, further promoting oxidative processes. Therefore, PDT serves as a source of H_2_O_2_ to sustain Fenton‐like reactions, whereas ^1^O_2_ directly oxidizes membrane lipids. Taken together, cuproptosis and PDT resulted in significant lipid ROS production, leading to tumor cell death.

(1) $${\mathrm{Cu}}^{+}+H_{2}O_{2}\to {\mathrm{Cu}}^{2+}+•\mathrm{OH}+{\mathrm{OH}}^{-}$$

(2) $${\mathrm{Cu}}^{2+}+H_{2}O_{2}\to {\mathrm{Cu}}^{+}+•{\mathrm{OH}}_{2}+{\mathrm{OH}}^{-}$$

## 3 Emerging NP‐Mediated Delivery System for Synergistic Cancer Therapy of Cuproptosis and PDT

**FIGURE 2:**
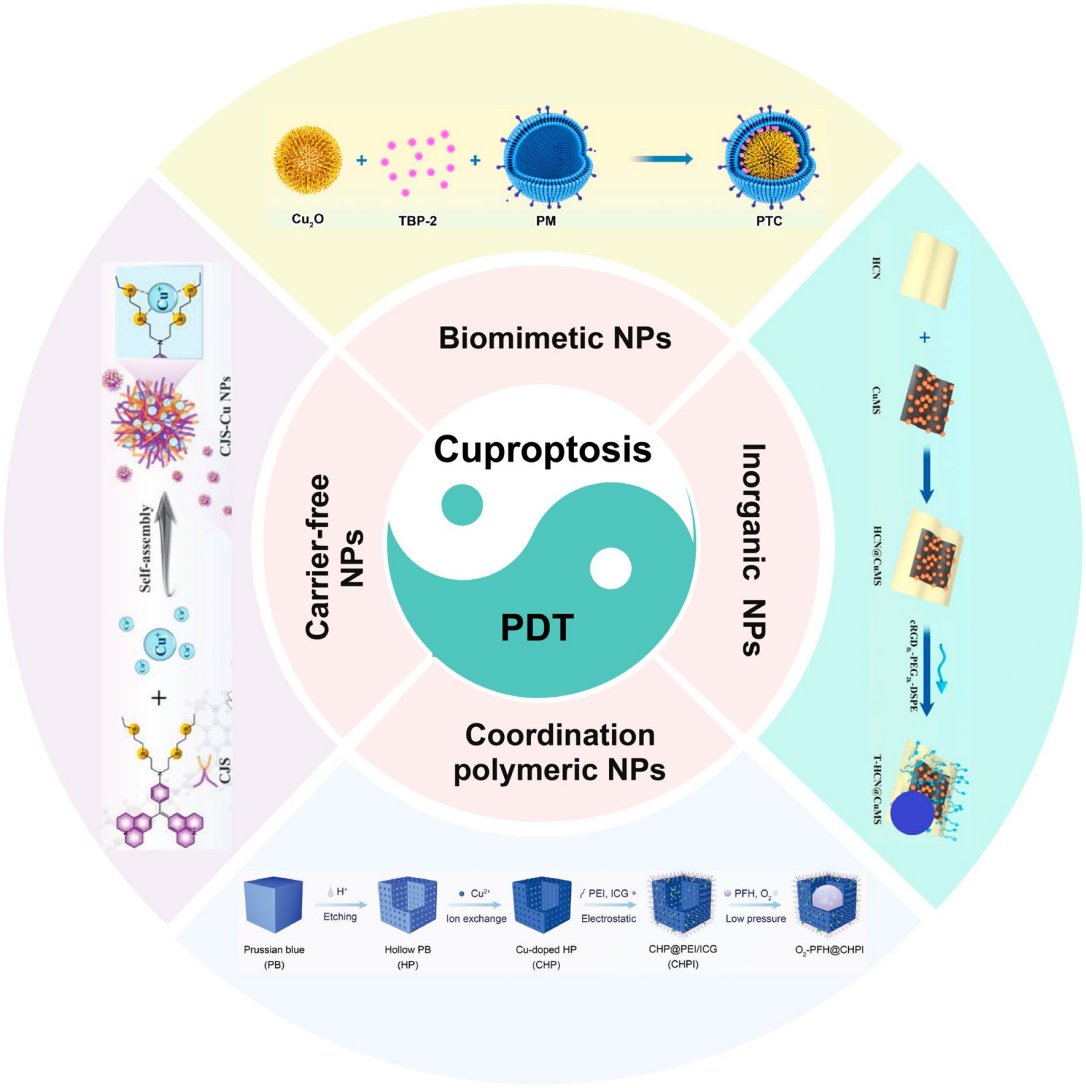
Schematic illustration of emerging NP‐assisted cuproptosis and PDT for synergistic cancer therapy. Created with BioRender.com.

Over the past few decades, nanotechnology has witnessed significant advancements, and various NPs such as biomimetic materials, metal‐organic frameworks (MOFs), inorganics, and carrier‐free NPs have been widely utilized as platform vectors for delivering PSs to tumors through active and passive targeting mechanisms [43]. The development of nanomedical drug delivery systems has improved drug solubility and circulation time, enabling localized accumulation at lesion sites while minimizing systemic side effects [44, 45, 46]. The NP treatment strategy combines the biochemical properties of cuproptosis with PDT to further disrupt the cellular redox balance and synergistically enhance its anticancer efficacy (Figure 2).

### 3.1 Cell‐Based Biomimetic NPs for Synergistic Therapy

Most NPs are synthetic, and as a result, the body recognizes them as foreign substances and may block drugs from entering the body. The “foreign” component of the NP causes it to be devoured by phagocytes, which can quickly be cleared by the immune system. To overcome this obstacle, extensive research is underway to develop biomimetic NPs that mimic cellular function [47]. The half‐life of platelets in circulation is approximately 30 h. This extended circulation duration can be partially attributed to the presence of CD47 (cluster of differentiation 47), a protein known as a “self‐marker,” which interacts with signal‐regulatory protein alpha on immune cells to inhibit their clearance [48, 49]. Consequently, platelet membrane‐coated NPs (PNPs) prolong the circulation time, facilitating passive targeted drug delivery. Platelets also express a unique set of surface receptors that dynamically adhere to damaged tumor cells, vascular systems, and pathogenic bacteria [50, 51, 52, 53]. Furthermore, compared with tumor cell membranes or leukocyte membranes, platelets are more easily obtainable, and the preparation of platelet vesicles (PVs) is less complex [54]. The broad and dynamic biointerfacing capabilities of PNPs make them attractive drug carriers for targeted delivery.

**FIGURE 3:**
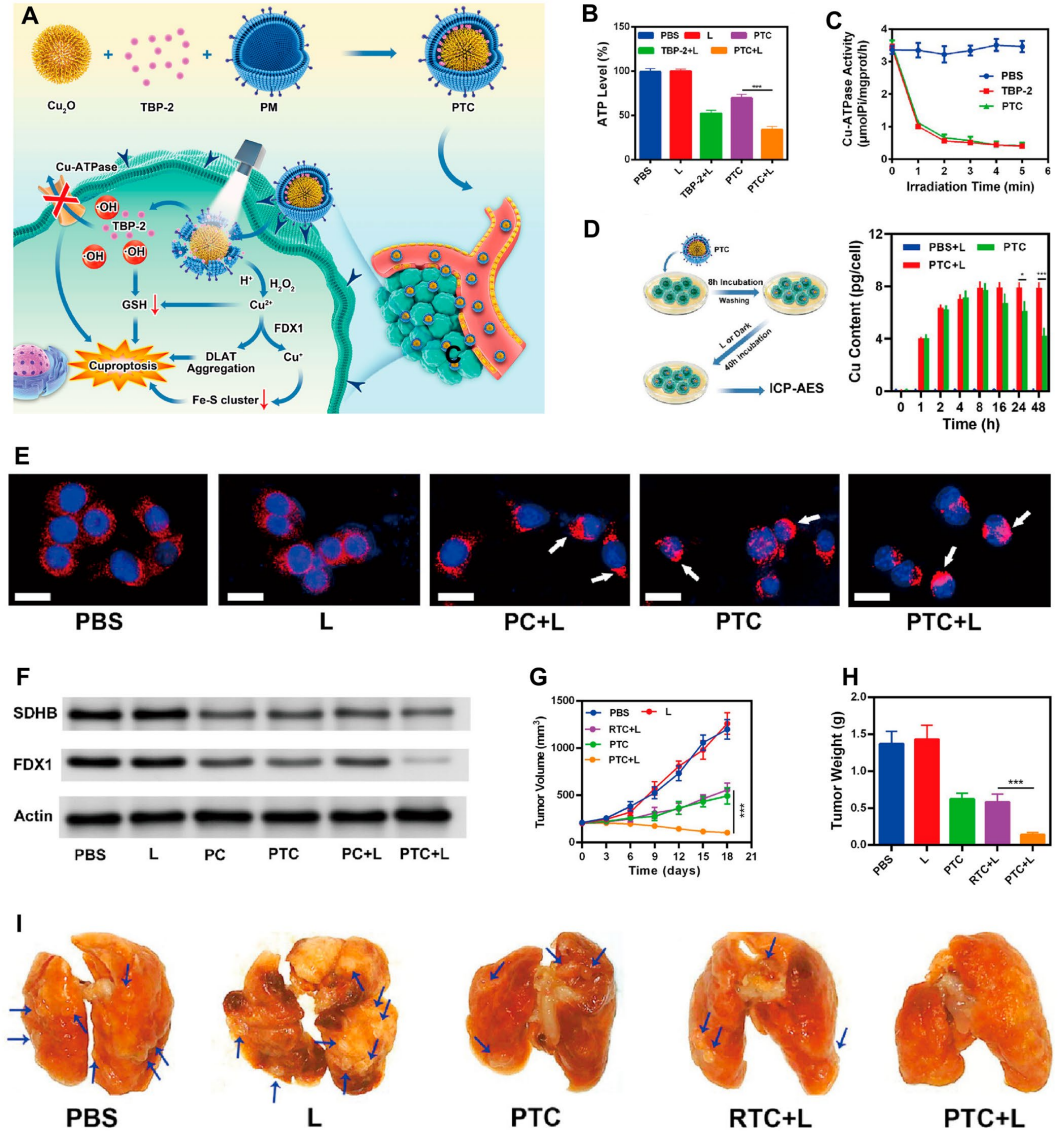
(A) Schematic illustration of biomimetic system PTC for tumor cuproptosis/PDT synergistic therapy. (B) Intracellular ATP levels of 4T1 after different treatment. (C) Cu‐ATPase activity changes after specified treatment under white light irradiation. (D) Schematic illustration and results of measuring intracellular Cu content during the treatment period. (E) DLAT fluorescence images of cancer cells after the indicated treatment. (F) Fe–S cluster protein expression in cancer cells after the indicated treatments. (G, H) Evolution of the tumor volume/weight in mice with different treatments. (I) The collected lungs in mice with different treatments. 4T1, mouse‐derived breast cancer cell line; ATP, adenosine triphosphate; DLAT, dihydrolipoamide S‐acetyltransferase; FDX1, ferredoxin 1; GSH, glutathione; ICP‐AES, inductively coupled plasma—atomic emission spectroscopy; PC, PM‐coated Cu_2_O without TBP‐2; PM, platelet membrane; PTC, platelet membrane‐coated Cu_2_O/TBP‐2 biomimetic cuproptosis sensitization system; RTC, red cell membrane‐coated Cu_2_O/TBP‐2 biomimetic cuproptosis sensitization system; TBP‐2, thioflavin B‐Photosensitizer 2. Reprinted from Ref. [55] with permission. Copyright (2023) the American Chemical Society. *** p‐value < 0.001, which is considered to be highly significant.

Tang et al. developed a cuproptosis‐sensitized system (platelet membrane‐coated Cu2O/TBP‐2 biomimetic cuproptosis sensitization system, PTC) composed of platelet membrane‐coated oxidized Cu NPs (Cu_2_O)/TBP‐2 for the simultaneous induction of tumor cuproptosis (Figure 3A) [55]. The TBP‐2 PS is an aggregation‐induced emission (AIE) compound that can be used for both Type I and Type II PDTs, with excellent water solubility and target affinity for tumor cell membranes [56]. In this study, PTC rapidly released bivalent Cu ions from tumor cells under the action of hydroxyl radicals (•OH) and hydrogen peroxide (H_2_O_2_), whereas TBP‐2 was released and quickly anchored under white light irradiation. After PTC + L treatment, the level of triphosphate (ATP) dropped significantly, indicating that PTC inhibited ATP generation (Figure 3B). This may be attributed to the mitochondrial damage caused by ROS. Inductively coupled plasma–atomic emission spectroscopy results showed that PTC‐mediated PDT inhibited the excretion of Cu ions in tumor cells (Figure 3D). On the one hand, this is due to cuproptosis, which damages mitochondrial function and reduces ATP content in tumor cells, thereby decreasing Cu ion excretion. On the other hand, TBP‐2‐mediated PDT generated large amounts of hydroxyl radicals, destroyed cell membranes, consumed cellular GSH, and lowered the activity of the Cu‐ATP enzyme (Figure 3C), thus inhibiting cellular Cu excretion. In addition, Cu ions directly bind to the lipoyl component of the TCA cycle, leading to the aggregation of fatty acylated proteins and loss of iron–sulfur proteins (Figure 3E,F). PTC demonstrated remarkable therapeutic efficacy in a 4 T1 breast cancer mouse model and effectively inhibited breast cancer‐associated lung metastasis. Meanwhile, by inducing cell membrane damage and GSH consumption through Type I photodynamic action, PTC significantly increased the incidence of cuproptosis in the tumor cells (Figure 3G–I).

### 3.2 Coordination Polymer‐Based NPs for Synergistic Therapy

**FIGURE 4:**
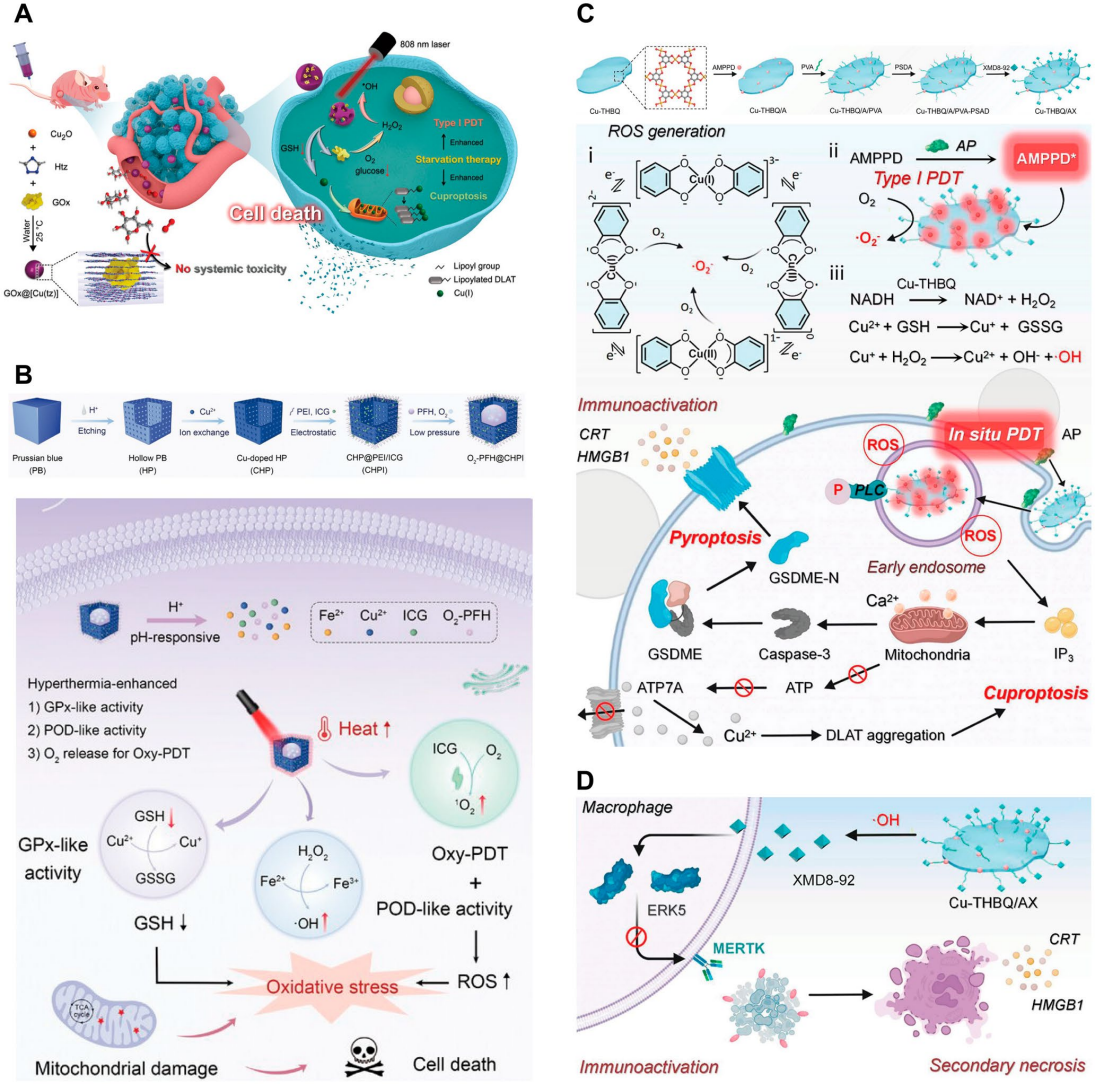
Coordination polymer as nanocarriers for synergistic cancer therapy. (A) Schematic illustration of the synthesis of nonporous GOx@[Cu(tz)] and their mechanism (Gox, glucose oxidase; Htz, 1,2,4‐triazole). Reprinted from Ref [22] with permission. Copyright (2022) WILEY. (B) Schematic illustration of the synthesis of O_2_‐PFH@CHPI nanozymes and their mechanism (GPx‐like, glutathione peroxidase‐like; GSH, glutathione; GSSG, oxidized glutathione; Oxy‐PDT, oxygen self‐enriching PDT; POD‐like, peroxidase‐like; ROS, reactive oxygen species). Reprinted from Ref [58] with permission. Copyright (2024) WILEY. (C) Schematic illustration of the synthesis of Cu‐THBQ/AX and the mechanism of cuproptosis/PDT synergistic cancer therapy. (D) Release of XMD8‐92 triggered by •OH effectively inhibits macrophage phagocytosis of apoptotic cancer cells, converting immunosuppressive cell death into proinflammatory secondary necrosis (AMPPD, adenosine 5′‐monophosphate propyl disulfide; AP, antioxidant protein; ATP7A, Cu‐transporting ATPase 7A; CRT, calreticulin; Cu‐THBQ/AX, Cu‐tetrahydroxybenzoquinone nanosized metal–organic framework; PVA, poly(vinyl alcohol); Cu‐THBQ, Cu‐tetrahydroxybenzoquinone; ERK5, extracellular signalregulated kinase 5; GSDME, cleavage of gasdermin‐E; GSDME‐N, gasdermin E N‐terminal domain; HMGB1, high mobility group box 1 protein; IL‐10, immunosuppressive cytokine interleukin 10; IP_3_, intracellular 1,4,5‐triphosphate; MERTK, MER protooncogene tyrosine kinase; NADH, nicotinamide adenine dinucleotide; PSDA, 2,2′‐[propane‐2,2‐diylbis(thio)]diacetic acid; XMD8‐92, extracellular signalregulated kinase 5 inhibitor). Reprinted from Ref [59] with permission. Copyright (2024) the American Chemical Society.

Coordination polymers (CPs) possess intrinsic metal ions and organic ligands that enable their use in the encapsulation and surface functionalization of various drugs and biomolecules to enhance their biocompatibility and targeting efficacy [57]. Nonporous coordination polymers can achieve controlled drug release by designing their structures and modifying their surfaces. For example, the drug release rate can be controlled by the temperature, pH, or other external stimuli. Pan et al. probed a nonporous Cu (I) 1,2,4‐triazole acid salt ([Cu(tz)]) coordination polymer nanoplatform GOx@[Cu(tz)] to potentiate cuproptosis and PDT during starvation (Figure 4A) [22]. When stimulated by high levels of GSH, glucose oxidase (GOx) is specifically released into the tumor cells, catalyzing glucose oxidation, causing increased H_2_O_2_ levels, and activating the hypoxia‐tolerant Type I PDT efficacy of GOx@[Cu(tz)]. A large amount of •OH was generated by the Fenton‐like reaction of Cu^+^/Cu^2+^. At the same time, the consumption of GSH and glucose led to increased sensitivity of tumor cells to cuproptosis induced by GOx@[Cu(tz)], as well as marked aggregation of lipoylated DLAT. This new type of NPs ultimately induced 92.4% tumor growth suppression through synergistic action, with high specificity and low systemic toxicity, thereby improving anticancer efficacy and safety.

Porous CPs, known as MOFs, integrate enzymes through postmodification or in situ encapsulation owing to their composition, structure, metal nodes, ligands, and morphology, and are widely used in preparing functional nanoporous materials [60, 61, 62, 63]. Prussian blue (PB) is a MOF composed of two octahedrally coordinated iron centers that exhibits high biocompatibility and has received approval from the US Food and Drug Administration for clinical applications in the treatment of radioactive poisoning [64]. In the realm of nanomedicine, PB‐based NPs exhibit biodegradability in a mildly acidic TME, allowing them to be metabolized and excreted by the body, avoiding potential long‐term toxicity and ensuring biocompatibility. It has been reported that PB NPs can serve as a new type of PS, effectively producing ^1^O_2_ for cancer treatment through energy transfer induced by near‐infrared radiation [65]. Xie et al. constructed Cu‐doped hollow PB (CHPB) NPs loaded with the PS indomyanine green (ICG) and O_2_‐saturated perfluorohexane (PFH) to induce PDT and cuproptosis (Figure 4B) [58]. Owing to PB's specific photothermal conversion properties [66, 67], CHP triggers O_2_ release under 808 nm laser irradiation, thereby enhancing ICG‐mediated PDT and facilitating the accumulation of ROS. Iron (Fe) ions in the PB can coordinate the catalysis of the Fenton reaction, deplete endogenous GSH, and induce oxidative stress. Concurrently, an elevated concentration of Cu ions induces DLAT aggregation and results in Fe–S cluster protein loss, leading to synchronized cuproptosis treatment. In this study, Cu‐induced oxidative stress disrupted mitochondrial metabolism and further disturbed the redox balance within the cells, thus realizing a synergistic therapeutic effect.

Recently, Zhao et al. designed a Cu‐tetrahydroxybenzoquinone (Cu‐THBQ/AX) nano‐sized MOF capable of co‐delivering 3‐(2‐spiroadamantyl)‐4‐methoxy‐4‐(3‐phosphoryloxy)‐phenyl‐1,2‐dioxetane (AMPPD, an alkaline phosphatase substrate) and XMD8‐92 (an inhibitor of extracellular signal‐regulated kinase 5) to suppress immunosuppressive tumors (Figure 4C) [59]. Cu‐THBQ generates •O_2_^−^ and •OH by oxidizing O_2_/H_2_O_2_ via a semiquinone radical and a Fenton‐like reaction, respectively. AMPPD also produces strong chemiluminescence (CL) in response to the overexpression of alkaline phosphatase (AP) on tumor cell surfaces, activating the Type I photodynamic process of Cu‐THBQ in situ. The accumulation of Cu ions led to the polymerization of DLAT. The generation of •OH also initiated the release of XMD8‐92, thereby effectively inhibiting macrophage autophagy and transforming the immunosuppressive apoptosis of cancer cells into inflammatory secondary necrosis (Figure 4D).

### 3.3 Inorganic‐Based NPs for Synergistic Therapy

**FIGURE 5:**
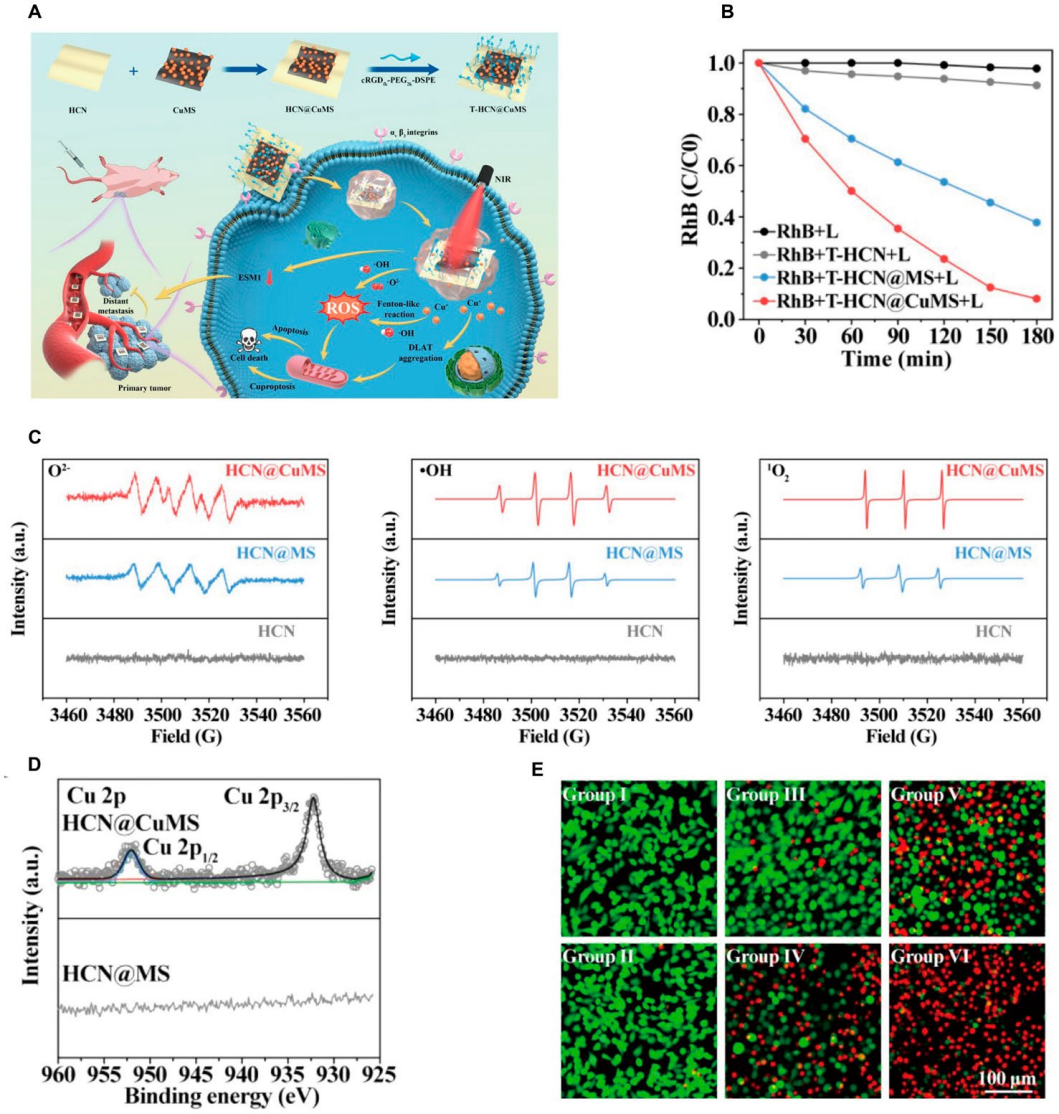
(A) Schematic illustration of the synthesis of T‐HCN@CuMS Nanoagent and their mechanism. (B) ROS produced by photocatalytic reaction was tested by Rhodamine B degradation assay in vitro. (C) ESR analysis of three typical ROS generation performance of different NPs under light irradiation. (D) High‐resolution XPS spectra of Cu 2p in HCN@CuMS and HCN@MS nanoheterojunction. (E) Live/dead staining of 143B cells after different treatments. (CuMS, Cu‐loaded molybdenum bisulfide nanosheets; ESM1, endothelial cellspecific molecule 1; ESR, electron spin resonance; HCN, heterogeneous carbon nitride nanosheets; HCN@CuMS, heterogeneous carbon nitride nanosheets @ Cu‐loaded metallic molybdenum bisulfide nanosheets; HCN@MS, heterogeneous carbon nitride nanosheets @ metallic molybdenum bisulfide nanosheets; NIR, near‐infrared; RhB, rhodamine B; ROS, reactive oxygen species; T‐HCN@CuMS, targeting nanoheterojunction; XPS, X‐ray photoelectron spectroscopy). Reprinted from Ref. [70] with permission. Copyright (2023) the American Chemical Society.

Graphitized carbon nitride (g‐C3N4) material is an innovative fluorescent polymer material with a dominant C and N atomic structure [68]. Carbon nitride materials have attracted widespread attention because of their wear resistance, high rigidity, chemical inertness, stable electron field emission, broadband optical transparency, and good biocompatibility [69]. Dai et al. constructed Cu‐loaded nanostructures (HCN@CuMS) from heterogeneous carbon nitride nanosheets (HCN) and Cu‐carrying molybdenum bisulfide nanosheets (CuMS), and modified them with cRDG‐PEG‐DSPE for the targeted treatment of highly malignant osteosarcoma (Figure 5A) [70]. The heterogeneous structure and Cu ion doping significantly improve the ROS production performance of HCN@CuMS in response to near‐infrared photocatalysis (Figure 5B,C). Additionally, a large number of Cu ions adsorbed on the surface of molybdenum disulfide exist in the form of monovalent Cu, which effectively catalyzes the Fenton‐like reaction and induces cuproptosis (Figure 5D,E). This method skillfully integrates the characteristics of chemocatalysis and Cu‐induced cell death, eliminates the step of Cu^2+^ reduction to Cu^+^, and greatly improves the efficiency of treatment.

### 3.4 Carrier‐Free NPs for Synergistic Therapy

**FIGURE 6:**
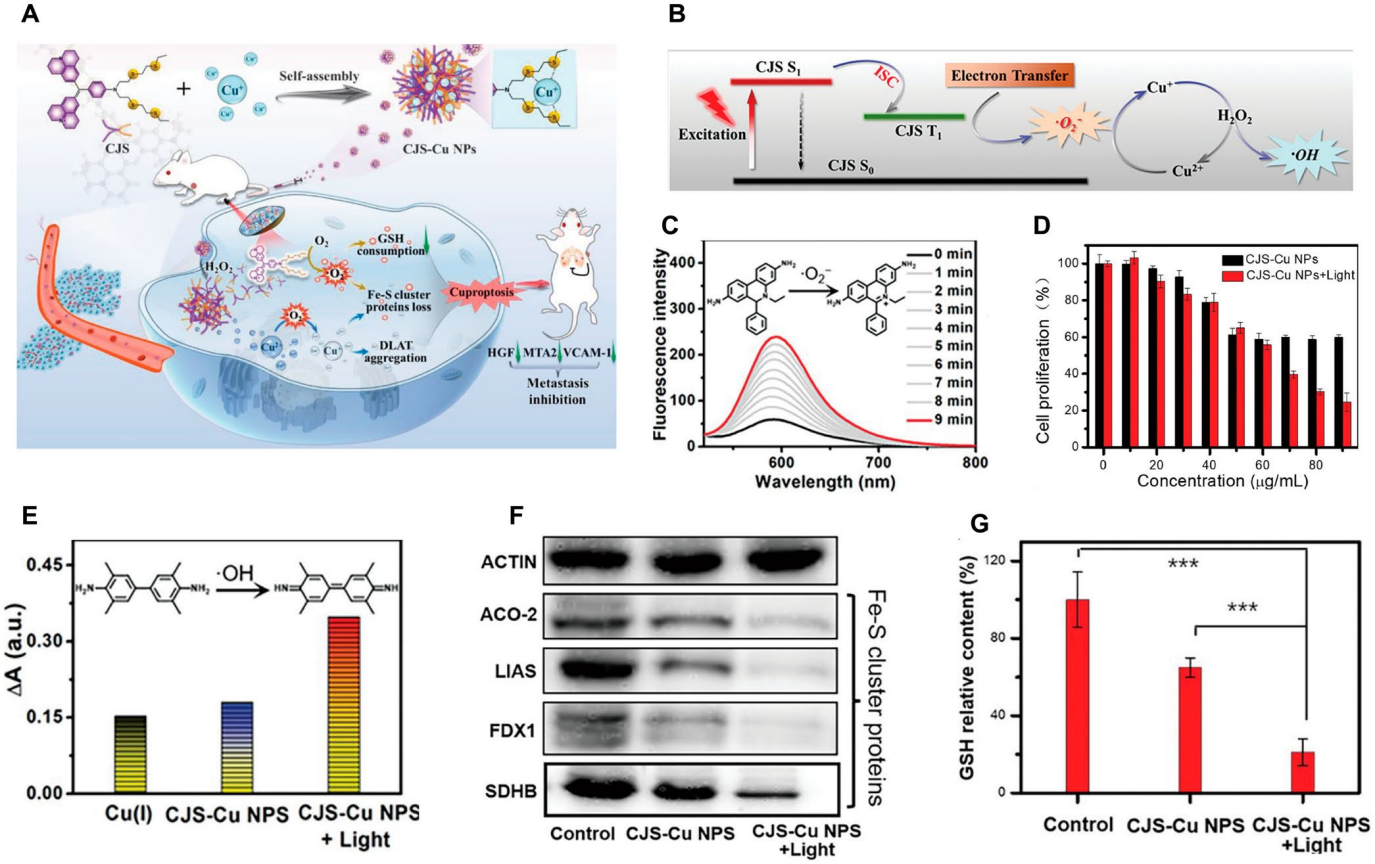
(A) Schematic illustration of the synthesis of CJS‐Cu NPs and their mechanism. (B) ROS production pathway of CJS‐Cu NPs. (C) The time‐dependent fluorescence intensity of the CJS‐Cu NPs under light irradiation indicates the generation of •O2−. (D) The cell proliferation was evaluated after treatment with different concentrations of CJS‐Cu NPs under various lighting conditions, and exhibited high cell‐killing efficacy under photoirradiation. (E) The relative TMB absorbance upon the addition of CJS‐Cu NPs and Cu+ with different lighting conditions. (F) Western blot analysis on the expressions of LIAS, ACO‐2, and FDX1. (G) Relative GSH contents in 4T1 cells after different treatments. ACO‐2, aconitase 2; DLAT, dihydrolipoamide S‐acetyltransferase; FDX1, ferredoxin 1; GSH, glutathione; HGF, hepatocyte growth factor/scatter factor; LIAS, lipoic acid synthase; MTA2, metastasis‐associated protein 2; ROS, reactive oxygen species; S0, singlet ground state; S1, singlet first excited state; SDHB, Succinate Dehydrogenase Subunit B; T1, triplet first excited state; TMB, 3,3,5,5′‐Tetramethylbenzidine; VCAM‐1, vascular cell adhesion molecule‐1. Reprinted from Ref. [72] with permission. Copyright (2023) WILEY. *** p‐value < 0.001, which is considered to be highly significant.

Carrier‐free nanomaterials have garnered significant attention in clinical translation owing to their exceptional drug‐loading capacity, facile synthesis methodology, and excellent biocompatibility [71]. Peng et al. successfully prepared uniformly distributed CJS‐Cu NPs by introducing Cu^+^ into type I photosensitized CJS using a simple self‐assembly method (Figure 6A) [72]. In this study, under light exposure, CJS‐Cu NPs produced •O_2_^−^, which initiated PDT (Figure 6B,C). Notably, after the release of Cu^2+^ ions in the presence of H_2_O_2_, the •O_2_^−^ generated by CJS‐Cu NPs can effectively reduce Cu^2+^ to Cu^+^, promoting cuproptosis (Figure 6B,D). Furthermore, Cu^+^ ions can trigger Fenton‐like reactions and produce Cu^2+^ and •OH, increasing ROS levels and leading to iron–sulfur cluster protein and GSH consumption (Figure 6E–G). These processes further enhance Cu‐induced cell death in cancer cells.

**FIGURE 7:**
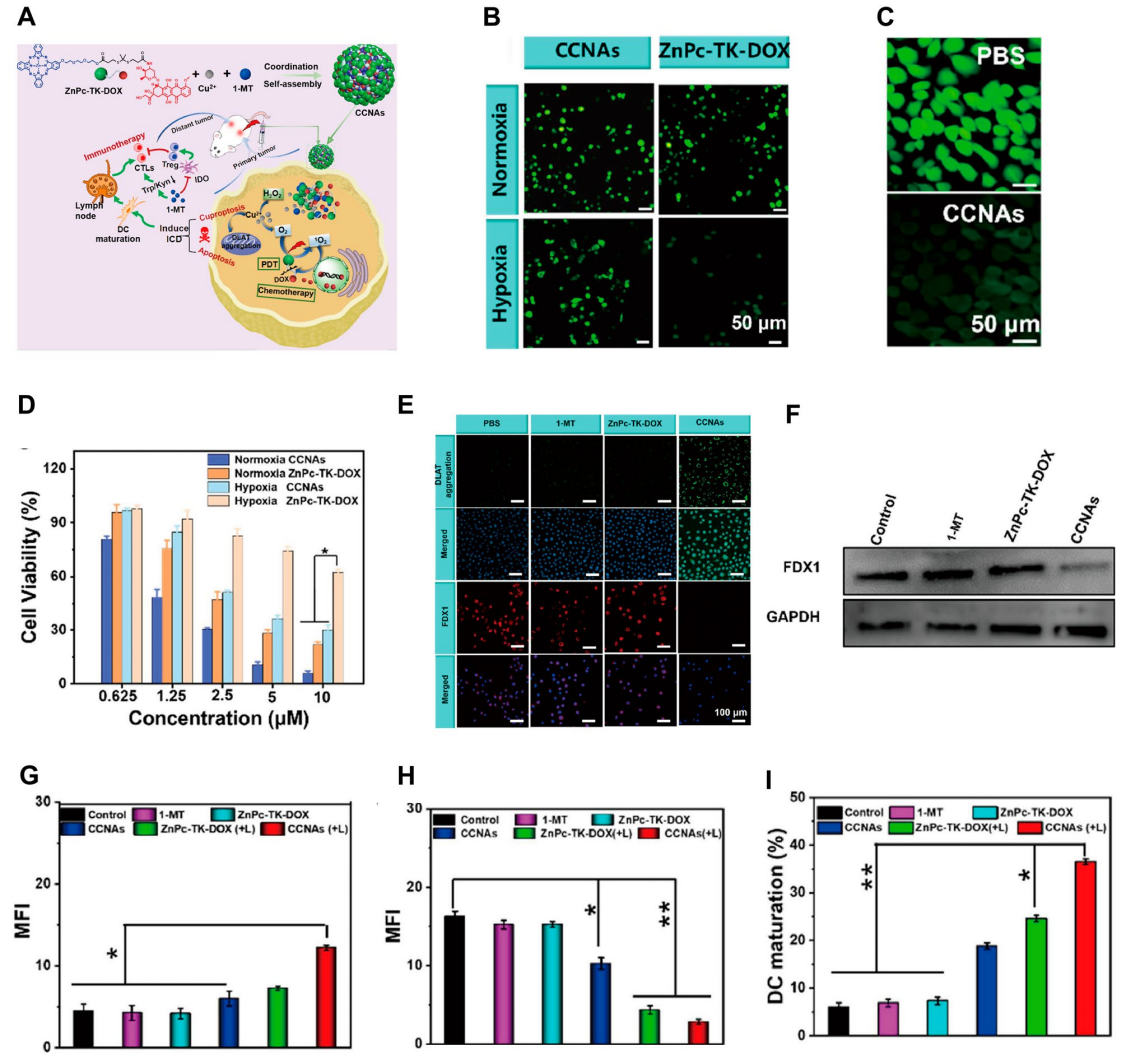
(A) Schematic illustration of the fabrication of CCNAs and their mechanism. (B) Fluorescence images of DCFHDA‐stained PC‐3 cells after different treatments in normoxia and hypoxia. (C) H_2_O_2_ staining images of PC‐3 cells after CCNA treatment. (D) The viability of PC‐3 cells following various treatments. (E) Immunofluorescence staining of DLAT aggregation and FDX1 in PC‐3 cells in different groups. (F) Western blot analysis of FDX1 expression after different treatments. (G) The mean fluorescence intensity (MFI) of CRT surface exposure on PC‐3 cells after the indicated treatments. (H) The mean fluorescence intensity (MFI) of CRT surface exposure on PC‐3 cells after the indicated treatments. (I) The MFI of HMGB1 released after the indicated treatments in PC‐3 cells. CCNAs, Cu‐coordinated nanoassemblies; CTLs, cytotoxic T lymphocytes; CRT, calreticulin; CFHDA, 2′,7′‐dichlorodihydrofluorescein diacetate; DLAT, dihydrolipoamide s‐acetyltransferase; DOX, doxorubicin; FDXI, ferredoxin 1; GAPDH, glyceraldehyde 3‐phosphate dehydrogenase; ICD, immunogenic cell death; IDO, ndoleamine 2,3‐dioxygenase; PC‐3, prostate cancer cell line 3; TK, thioketal; Trp/Kyn, tryptophan/kynurenine ratio; ZnPc, zinc phthalocyanine; ZnPc‐TK‐DOX, a prodrug composed of the photosensitizer ZnPc and the chemotherapy drug DOX linked by thioketone TK). Reprinted from Ref. [24] with permission. Copyright (2024) ELSEVIER. A single asterisk (*) is used to denote a p‐value of less than 0.05, which is considered to be statistically significant. This means that there is less than a 5% probability that the observed difference occurred by chance. A double asterisk (**) is used to denote a p‐value of less than 0.01, which is considered to be highly statistically significant. This indicates that there is less than a 1% probability that the observed difference is due to random variation.

In addition, the self‐assembly of a PS‐chemotherapeutic prodrug with Cu is an effective strategy for tumor treatment. Li et al. developed a prodrug consisting of a PS (Zinc Phthalocyanine, ZnPc) conjugated to DOX (doxorubicin) via a ROS‐responsive thioketal (TK) linker (Figure 7A) [24]. In this study, laser irradiation was employed to activate PDT and generate ^1^O_2_, leading to rapid rupture of the TK bond and the subsequent release of DOX. Cu^2+^ ions facilitated the Fenton‐like reaction of intracellular H_2_O_2_ into O_2_, thus enhancing the efficacy of PDT in hypoxic cancer cells (Figure 7B–D). Furthermore, Cu^2+^/Cu^+^ ions induced lipoylated DLAT aggregation, leading to cuproptosis (Figure 7E,F). Importantly, enhanced Cu‐induced apoptosis triggers an immunogenic cell death (ICD) response (Figure 7G–I).

## 4 Conclusions and Future Perspectives

Cancer is a widespread and devastating disease, recognized as the leading cause of mortality worldwide, and is responsible for approximately 13% of all annual deaths [73, 74]. Despite substantial advancements in both fundamental and clinical cancer research, numerous treatments continue to pose challenges and exhibit limited efficacy. Researchers are continuously exploring innovative strategies that integrate drugs with diverse therapeutic mechanisms to investigate combination therapies. Cuproptosis, a novel programmed cell death regulated by Cu ions, was proposed in 2022, which not only solves the issue of resistance caused by cell apoptosis but also avoids the potential risks of necrosis [75, 76]. Recent investigations have indicated that integrating cuproptosis with PDT in oncological treatment may offer significant advantages, particularly when combined with nanotechnology, to facilitate tumor cell‐specific uptake and controlled release. In this review, we discuss several advanced multifunctional NPs capable of combining cuproptosis and PDT, demonstrating promising therapeutic potential compared to individual applications of cuproptosis and PDT alone.

Cuproptosis is a process of cell apoptosis triggered by Cu ions. Cu ions bind to sulfur‐containing proteins in the mitochondria, disrupting the stability of iron–sulfur clusters, leading to mitochondrial metabolic disorders and the generation of oxidative stress, thereby inducing cell apoptosis. Cu‐based nanomaterials can produce ROS and O_2_ through Fenton‐like reactions, enhancing the effects of PDT and alleviating tumor hypoxia. By leveraging photothermal effects to trigger the release of Cu ions, further promotion of cuproptosis and apoptosis can be achieved, increasing the killing efficiency of tumor cells. The synergistic effect of cuproptosis and PDT can enhance the anticancer effect by dual induction of apoptosis. On the one hand, the accumulation of Cu ions leads to mitochondrial dysfunction; on the other hand, the reactive O_2_ produced by PDT exacerbates oxidative stress, and both act together on apoptosis. Both therapies involve oxidative stress and the destruction of mitochondrial function, so they may achieve a synergistic effect through commonly regulated signaling pathways. In addition, as a form of regulated cell death (RCD) caused by metal ion homeostasis disorders, Cu^+^/Cu^2+^ has superior catalytic activity and pH adaptability compared to Fe^2+^, indicating that Cu‐based nanomedicines may have greater potential for clinical application.

Despite progress in leveraging the synergistic effects between cuproptosis and PDT, there are still challenges and issues. Firstly, further research is needed to clarify the regulatory mechanisms of cuproptosis and its related signaling pathways [27, 77], to deepen our understanding of this synergistic therapy, and to guide the design of more effective anticancer nanomaterials. Secondly, an increasing number of bioinformatics studies suggest that cuproptosis has the potential to enhance immunotherapy. However, the specific regulatory mechanisms that link cuproptosis to improved outcomes in immunotherapy are still unclear. Clarifying this association could bring significant benefits to patients with cancer. Thirdly, in cancer treatment, cuproptosis is a double‐edged sword, capable of killing cancer cells but also potentially causing Cu poisoning and damaging normal cells [78, 79]. Therefore, how to strengthen drug targeting, optimize dosage, and ensure long‐term biosafety has become an urgent issue to be resolved. Fourthly, GSH overexpressed in the TME can act as a Cu chelator, removing excess Cu ions and protecting tumor cells from cuproptosis [22], while also reducing the ROS produced by PDT, weakening its effect. Therefore, developing GSH inhibition strategies is crucial for driving cuproptosis and PDT simultaneously [80, 81]. Fifthly, as Type II PSs are highly O_2_‐dependent, the hypoxic TME caused by O_2_ consumption can significantly affect the sustained effect of PDT [82, 83]. Therefore, developing new PSs, utilizing Type I PSs, or designing O_2_‐saving photodynamic therapies can effectively overcome the impact of tumor hypoxia on PDT effects, providing new ideas and methods to improve PDT treatment outcomes. Finally, given that Cu participates in the TCA cycle within the mitochondria to induce cuproptosis, enhancing the mitochondrial targeting of drugs to achieve efficient treatment at low doses and reduce side effects is worth further exploration.

In summary, the synergistic therapeutic strategy of cuproptosis and PDT in the field of cancer treatment shows great potential, but to achieve its clinical application, extensive and in‐depth research on the above challenges is still required. The synergistic treatment of PDT and cuproptosis is complementary in tumor therapy; however, comprehensive research and strategy optimization are essential. Through a comprehensive study of its mechanisms of action and optimization of treatment plans, we are confident that this treatment strategy will provide new opportunities for humanity to overcome cancer.

## Author Contributions

**Junrui Zhang:** conceptualization; writing – original draft; validation; visualization; project administration; resources; data curation. **Anren Zhang:** data curation; software; methodology. **Yibing Guo:** validation; formal analysis. **Guoliang Miao:** visualization; validation; project administration. **Shengchang Liang:** investigation; visualization; formal analysis. **Jie Wang:** conceptualization; writing – review and editing; software; supervision. **Junhong Wang:** writing – review and editing; funding acquisition; project administration.

## Consent

The authors have nothing to report.

## Conflicts of Interest

The authors declare no conflicts of interest.

## Data Availability

Data sharing is not applicable to this article as no new data were created or analyzed in this study.

## References

[1] F. Bray , M. Laversanne , H. Sung , et al., “Global Cancer Statistics 2022: GLOBOCAN Estimates of Incidence and Mortality Worldwide for 36 Cancers in 185 Countries,” CA: A Cancer Journal for Clinicians 74, no. 3 (2024): 229–263. 38572751 10.3322/caac.21834

[2] O. I. Kit , O. V. Katelnitskaya , A. A. Maslov , et al., “Combined Surgeries for Retroperitoneal Tumors,” Journal of Clinical Oncology 38, no. 15_suppl (2020): e23579.

[3] H. Chen , Z. Han , Q. Luo , et al., “Radiotherapy Modulates Tumor Cell Fate Decisions: A Review,” Radiation Oncology 17, no. 1 (2022): 196. 36457125 10.1186/s13014-022-02171-7 PMC9714175

[4] C. D'alterio , S. Scala , G. Sozzi , et al., “Paradoxical Effects of Chemotherapy on Tumor Relapse and Metastasis Promotion,” Seminars in Cancer Biology 60 (2020): 351–361. 31454672 10.1016/j.semcancer.2019.08.019

[5] Y. Wang , D. Wang , Y. Zhang , et al., “Tumor Microenvironment‐Adaptive Nanoplatform Synergistically Enhances Cascaded Chemodynamic Therapy,” Bioactive Materials 22 (2023): 239–253. 36254272 10.1016/j.bioactmat.2022.09.025 PMC9550605

[6] T. C. Pham , V. N. Nguyen , Y. Choi , S. Lee , and J. Yoon , “Recent Strategies to Develop Innovative Photosensitizers for Enhanced Photodynamic Therapy,” Chemical Reviews 121, no. 21 (2021): 13454–13619. 34582186 10.1021/acs.chemrev.1c00381

[7] M. Wu , Q. Zhang , Y. Fang , et al., “Polylysine‐Modified MXene Nanosheets With Highly Loaded Glucose Oxidase as Cascade Nanoreactor for Glucose Decomposition and Electrochemical Sensing,” Journal of Colloid and Interface Science 586 (2021): 20–29. 33153715 10.1016/j.jcis.2020.10.065

[8] Y. Liu , P. Bhattarai , Z. Dai , and X. Chen , “Photothermal Therapy and Photoacoustic Imaging via Nanotheranostics in Fighting Cancer,” Chemical Society Reviews 48, no. 7 (2019): 2053–2108. 30259015 10.1039/c8cs00618k PMC6437026

[9] P. Lelièvre , L. Sancey , J. L. Coll , A. Deniaud , and B. Busser , “The Multifaceted Roles of Copper in Cancer: A Trace Metal Element With Dysregulated Metabolism, but Also a Target or a Bullet for Therapy,” Cancers (Basel) 12, no. 12 (2020): 3594. 33271772 10.3390/cancers12123594 PMC7760327

[10] D. Jia , L. Liu , W. Liu , J. Li , X. Jiang , and Y. Xin , “Copper Metabolism and Its Role in Diabetic Complications: A Review,” Pharmacological Research 206 (2024): 107264. 38876443 10.1016/j.phrs.2024.107264

[11] P. Tsvetkov , S. Coy , B. Petrova , et al., “Copper Induces Cell Death by Targeting Lipoylated TCA Cycle Proteins,” Science 375, no. 6586 (2022): 1254–1261. 35298263 10.1126/science.abf0529 PMC9273333

[12] C. Chen , C. Wu , J. Yu , et al., “Photodynamic‐Based Combinatorial Cancer Therapy Strategies: Tuning the Properties of Nanoplatform According to Oncotherapy Needs,” Coordination Chemistry Reviews 461 (2022): 214495.

[13] M. Zhang , Y. Zhao , H. Ma , Y. Sun , and J. Cao , “How to Improve Photodynamic Therapy‐Induced Antitumor Immunity for Cancer Treatment?,” Theranostics 12, no. 10 (2022): 4629–4655. 35832074 10.7150/thno.72465 PMC9254244

[14] N. Nombona , E. Antunes , W. Chidawanyika , P. Kleyi , Z. Tshentu , and T. Nyokong , “Synthesis, Photophysics and Photochemistry of Phthalocyanine‐ɛ‐Polylysine Conjugates in the Presence of Metal Nanoparticles Against *Staphylococcus aureus* ,” Journal of Photochemistry and Photobiology A: Chemistry 233 (2012): 24–33.

[15] M. Lan , S. Zhao , W. Liu , C. S. Lee , W. Zhang , and P. Wang , “Photosensitizers for Photodynamic Therapy,” Advanced Healthcare Materials 8, no. 13 (2019): 1900132. 10.1002/adhm.201900132 31067008

[16] M. Tavakkoli Yaraki , B. Liu , and Y. N. Tan , “Emerging Strategies in Enhancing Singlet Oxygen Generation of Nano‐Photosensitizers Toward Advanced Phototherapy,” Nano‐Micro Letters 14, no. 1 (2022): 123. 35513555 10.1007/s40820-022-00856-y PMC9072609

[17] D. Chen , Q. Xu , W. Wang , J. Shao , W. Huang , and X. Dong , “Type I Photosensitizers Revitalizing Photodynamic Oncotherapy,” Small 17, no. 31 (2021): e2006742. 34038611 10.1002/smll.202006742

[18] X. Xu , M. Chen , X. Lou , et al., “Sialic Acid‐Modified Mesoporous Polydopamine Induces Tumor Vessel Normalization to Enhance Photodynamic Therapy by Inhibiting VE‐Cadherin Internalization,” Chemical Engineering Journal 414 (2021): 128743.

[19] R. Alzeibak , T. A. Mishchenko , N. Y. Shilyagina , I. V. Balalaeva , M. V. Vedunova , and D. V. Krysko , “Targeting Immunogenic Cancer Cell Death by Photodynamic Therapy: Past, Present and Future,” Journal for Immunotherapy of Cancer 9, no. 1 (2021): e001926. 33431631 10.1136/jitc-2020-001926 PMC7802670

[20] J. Zhou , Q. Yu , J. Song , et al., “Photothermally Triggered Copper Payload Release for Cuproptosis‐Promoted Cancer Synergistic Therapy,” Angewandte Chemie (International Edition) 62, no. 12 (2023): e202213922. 36585379 10.1002/anie.202213922

[21] Y. N. Hao , W. X. Zhang , Y. R. Gao , Y. N. Wei , Y. Shu , and J. H. Wang , “State‐Of‐The‐Art Advances of Copper‐Based Nanostructures in the Enhancement of Chemodynamic Therapy,” Journal of Materials Chemistry B 9, no. 2 (2021): 250–266. 33237121 10.1039/d0tb02360d

[22] Y. Xu , S. Y. Liu , L. Zeng , et al., “An Enzyme‐Engineered Nonporous Copper(I) Coordination Polymer Nanoplatform for Cuproptosis‐Based Synergistic Cancer Therapy,” Advanced Materials 34, no. 43 (2022): e2204733. 36054475 10.1002/adma.202204733

[23] L. Han , Y. Wang , X. Huang , et al., “Specific‐Oxygen‐Supply Functionalized Core‐Shell Nanoparticles for Smart Mutual‐Promotion Between Photodynamic Therapy and Gambogic Acid‐Induced Chemotherapy,” Biomaterials 257 (2020): 120228. 32736257 10.1016/j.biomaterials.2020.120228

[24] W. Liang , C. Han , D. Zhang , et al., “Copper‐Coordinated Nanoassemblies Based on Photosensitizer‐Chemo Prodrugs and Checkpoint Inhibitors for Enhanced Apoptosis‐Cuproptosis and Immunotherapy,” Acta Biomaterialia 175 (2024): 341–352. 38122883 10.1016/j.actbio.2023.12.022

[25] P. Tsvetkov , S. Coy , B. Petrova , et al., “Copper Induces Cell Death by Targeting Lipoylated TCA Cycle Proteins,” Science 375 (2022): 1254–1261. 35298263 10.1126/science.abf0529 PMC9273333

[26] R. M. Mohammad , I. Muqbil , L. Lowe , et al., “Broad Targeting of Resistance to Apoptosis in Cancer,” Seminars in Cancer Biology 35 (2015): S78–S103. 25936818 10.1016/j.semcancer.2015.03.001 PMC4720504

[27] D. Tang , X. Chen , and G. Kroemer , “Cuproptosis: A Copper‐Triggered Modality of Mitochondrial Cell Death,” Cell Research 32, no. 5 (2022): 417–418. 35354936 10.1038/s41422-022-00653-7 PMC9061796

[28] J. Du , T. Shi , S. Long , et al., “Enhanced Photodynamic Therapy for Overcoming Tumor Hypoxia: From Microenvironment Regulation to Photosensitizer Innovation,” Coordination Chemistry Reviews 427 (2021): 213604.

[29] X. Zheng , W. Sun , M. Ju , J. Wu , H. Huang , and B. Shen , “A Chemical Biology Toolbox to Overcome the Hypoxic Tumor Microenvironment for Photodynamic Therapy: A Review,” Biomaterials Science 10, no. 17 (2022): 4681–4693. 35822831 10.1039/d2bm00776b

[30] R. Bevernaegie , B. Doix , E. Bastien , et al., “Exploring the Phototoxicity of Hypoxic Active Iridium(III)‐Based Sensitizers in 3D Tumor Spheroids,” Journal of the American Chemical Society 141, no. 46 (2019): 18486–18491. 31644286 10.1021/jacs.9b07723

[31] Y.‐Y. Wang , Y.‐C. Liu , H. Sun , and D. S. Guo , “Type I Photodynamic Therapy by Organic–Inorganic Hybrid Materials: From Strategies to Applications,” Coordination Chemistry Reviews 395 (2019): 46–62.

[32] J. Ni , Y. Wang , H. Zhang , J. Z. Sun , and B. Z. Tang , “Aggregation‐Induced Generation of Reactive Oxygen Species: Mechanism and Photosensitizer Construction,” Molecules 26, no. 2 (2021): 268. 33430513 10.3390/molecules26020268 PMC7827197

[33] B. Zheng , Y. Zhao , H. Li , et al., “Activatable Anticancer Photosensitizers,” Chemical Advances 30, no. 9 (2018): 1403–1414.

[34] D. Van Straten , V. Mashayekhi , H. S. De Bruijn , et al., “Oncologic Photodynamic Therapy: Basic Principles, Current Clinical Status and Future Directions,” Cancers (Basel) 9, no. 2 (2017): 19. 28218708 10.3390/cancers9020019 PMC5332942

[35] Z. Zhou , J. Song , L. Nie , and X. Chen , “Reactive Oxygen Species Generating Systems Meeting Challenges of Photodynamic Cancer Therapy,” Chemical Society Reviews 45, no. 23 (2016): 6597–6626. 27722328 10.1039/c6cs00271d PMC5118097

[36] T. A. Mishchenko , I. V. Balalaeva , M. V. Vedunova , and D. V. Krysko , “Ferroptosis and Photodynamic Therapy Synergism: Enhancing Anticancer Treatment,” Trends Cancer 7, no. 6 (2021): 484–487. 33640304 10.1016/j.trecan.2021.01.013

[37] J. An , S. Tang , G. Hong , et al., “An Unexpected Strategy to Alleviate Hypoxia Limitation of Photodynamic Therapy by Biotinylation of Photosensitizers,” Nature Communications 13, no. 1 (2022): 2225. 10.1038/s41467-022-29862-9 PMC9038921 35469028

[38] H. Huang , W. Xie , Q. Wan , et al., “A Self‐Degradable Conjugated Polymer for Photodynamic Therapy With Reliable Postoperative Safety,” Advanced Science 9, no. 4 (2022): e2104101. 34898054 10.1002/advs.202104101 PMC8811814

[39] Y. Sun , D. Zhao , G. Wang , et al., “Recent Progress of Hypoxia‐Modulated Multifunctional Nanomedicines to Enhance Photodynamic Therapy: Opportunities, Challenges, and Future Development,” Acta Pharmaceutica Sinica B 10, no. 8 (2020): 1382–1396. 32963938 10.1016/j.apsb.2020.01.004 PMC7488364

[40] V. D. Turubanova , I. V. Balalaeva , T. A. Mishchenko , et al., “Immunogenic Cell Death Induced by a New Photodynamic Therapy Based on Photosens and Photodithazine,” Journal for Immunotherapy of Cancer 7, no. 1 (2019): 350. 31842994 10.1186/s40425-019-0826-3 PMC6916435

[41] F. Sun , Q. Zhu , T. Li , et al., “Regulating Glucose Metabolism With Prodrug Nanoparticles for Promoting Photoimmunotherapy of Pancreatic Cancer,” Advanced Science 8, no. 4 (2021): 2002746. 33643795 10.1002/advs.202002746 PMC7887571

[42] A. D. Bokare and W. Choi , “Review of Iron‐Free Fenton‐Like Systems for Activating H_2_O_2_ in Advanced Oxidation Processes,” Journal of Hazardous Materials 275 (2014): 121–135. 24857896 10.1016/j.jhazmat.2014.04.054

[43] H. Abrahamse and M. R. Hamblin , “New Photosensitizers for Photodynamic Therapy,” Biochemical Journal 473, no. 4 (2016): 347–364. 26862179 10.1042/BJ20150942 PMC4811612

[44] P. Huang , X. Wang , X. Liang , et al., “Nano‐, Micro‐, and Macroscale Drug Delivery Systems for Cancer Immunotherapy,” Acta Biomaterialia 85 (2019): 1–26. 30579043 10.1016/j.actbio.2018.12.028

[45] P. Fu , J. Zhang , H. Li , M. Mak , W. Xu , and Z. Tao , “Extracellular Vesicles as Delivery Systems at Nano−/Micro‐Scale,” Advanced Drug Delivery Reviews 179 (2021): 113910. 34358539 10.1016/j.addr.2021.113910 PMC8986465

[46] Q. Fu , X. Zhang , J. Song , and H. Yang , “Plasmonic Gold Nanoagents for Cancer Imaging and Therapy,” View 2 (2021): 20200149.

[47] R. H. Fang , Y. Jiang , J. C. Fang , and L. Zhang , “Cell Membrane‐Derived Nanomaterials for Biomedical Applications,” Biomaterials 128 (2017): 69–83. 28292726 10.1016/j.biomaterials.2017.02.041 PMC5417338

[48] L. Yang , K. Zhang , D. Zheng , et al., “Platelet‐Based Nanoparticles With Stimuli‐Responsive for Anti‐Tumor Therapy,” International Journal of Nanomedicine 18 (2023): 6293–6309. 37954456 10.2147/IJN.S436373 PMC10637234

[49] Z. He , Y. Zhang , and N. Feng , “Cell Membrane‐Coated Nanosized Active Targeted Drug Delivery Systems Homing to Tumor Cells: A Review,” Materials Science & Engineering. C, Materials for Biological Applications 106 (2020): 110298. 31753336 10.1016/j.msec.2019.110298

[50] D. Lievens and P. Von Hundelshausen , “Platelets in Atherosclerosis,” Thrombosis and Haemostasis 106, no. 5 (2011): 827–838. 22012554 10.1160/TH11-08-0592

[51] X. Cai , L. Qiu , Z. Diao , L. Cai , T. Yin , and H. Pan , “Platelet‐Based Bioactive Systems Guided Precision Targeting and Immune Regulation for Cancer Therapy,” Nano Research 17, no. 9 (2024): 8269–8284.

[52] J. R. Fitzgerald , T. J. Foster , and D. Cox , “The Interaction of Bacterial Pathogens With Platelets,” Nature Reviews. Microbiology 4, no. 6 (2006): 445–457. 16710325 10.1038/nrmicro1425

[53] Y. He , R. Li , J. Liang , et al., “Drug Targeting Through Platelet Membrane‐Coated Nanoparticles for the Treatment of Rheumatoid Arthritis,” Nano Reseach 11 (2018): 6086–6101.

[54] D. Zhu , R. Ling , H. Chen , et al., “Biomimetic Copper Single‐Atom Nanozyme System for Self‐Enhanced Nanocatalytic Tumor Therapy,” Nano Reseach 15 (2022): 7320–7328.

[55] S. Ning , M. Lyu , D. Zhu , et al., “Type‐I AIE Photosensitizer Loaded Biomimetic System Boosting Cuproptosis to Inhibit Breast Cancer Metastasis and Rechallenge,” ACS Nano 17, no. 11 (2023): 10206–10217. 37183977 10.1021/acsnano.3c00326

[56] D. Zhu , J. Zhang , G. Luo , Y. Duo , and B. Z. Tang , “Bright Bacterium for Hypoxia‐Tolerant Photodynamic Therapy Against Orthotopic Colon Tumors by an Interventional Method,” Advanced Science 8, no. 15 (2021): e2004769. 34145986 10.1002/advs.202004769 PMC8336512

[57] S. Suárez‐García , R. Solórzano , R. Alibés , et al., “Antitumour Activity of Coordination Polymer Nanoparticles,” 441 (2021).

[58] X. Zhang , J. Zhu , S. Wang , et al., “A Copper/Ferrous‐Engineering Redox Homeostasis Disruptor for Cuproptosis/Ferroptosis co‐Activated Nanocatalytic Therapy in Liver Cancer,” Advanced Functional Materials 34 (2024): 2402022.

[59] Y. Liu , R. Niu , X. Zhang , et al., “Metal‐Organic Framework‐Based Nanovaccine for Relieving Immunosuppressive Tumors via Hindering Efferocytosis of Macrophages and Promoting Pyroptosis and Cuproptosis of Cancer Cells,” ACS Nano 18, no. 19 (2024): 12386–12400. 38699808 10.1021/acsnano.4c01518

[60] Z. X. Cai , Z. L. Wang , J. Kim , and Y. Yamauchi , “Hollow Functional Materials Derived From Metal‐Organic Frameworks: Synthetic Strategies, Conversion Mechanisms, and Electrochemical Applications,” Advanced Materials 31, no. 11 (2019): e1804903. 30637804 10.1002/adma.201804903

[61] M. D. J. Velásquez‐Hernández , M. Linares‐Moreau , E. Astria , et al., “Towards Applications of Bioentities@ MOFs in Biomedicine,” Coordination Chemistry Reviews 429 (2021): 213651.

[62] H. Zhang , X. Liu , Y. Wu , C. Guan , A. K. Cheetham , and J. Wang , “MOF‐Derived Nanohybrids for Electrocatalysis and Energy Storage: Current Status and Perspectives,” Chemical Communications 54, no. 42 (2018): 5268–5288. 29582028 10.1039/c8cc00789f

[63] B. Mohanty , S. Kumari , P. Yadav , P. Kanoo , and A. Chakraborty , “Metal‐Organic Frameworks (MOFs) and MOF Composites Based Biosensors,” Coordination Chemistry Reviews 519 (2024): 216102.

[64] X. Cai , K. Zhang , X. Xie , et al., “Self‐Assembly Hollow Manganese Prussian White Nanocapsules Attenuate Tau‐Related Neuropathology and Cognitive Decline,” Biomaterials 231 (2020): 119678. 31864019 10.1016/j.biomaterials.2019.119678

[65] B. Zhou , B. P. Jiang , W. Sun , et al., “Water‐Dispersible Prussian Blue Hyaluronic Acid Nanocubes With Near‐Infrared Photoinduced Singlet Oxygen Production and Photothermal Activities for Cancer Theranostics,” ACS Applied Materials & Interfaces 10, no. 21 (2018): 18036–18049. 29745229 10.1021/acsami.8b01387

[66] K. Zhang , J. Wu , X. Zhao , et al., “Prussian Blue/Calcium Peroxide Nanocomposites‐Mediated Tumor Cell Iron Mineralization for Treatment of Experimental Lung Adenocarcinoma,” ACS Nano 15, no. 12 (2021): 19838–19852. 34851083 10.1021/acsnano.1c07308

[67] L. Zhou , J. Chen , R. Li , et al., “Metal‐Polyphenol‐Network Coated Prussian Blue Nanoparticles for Synergistic Ferroptosis and Apoptosis via Triggered GPX4 Inhibition and Concurrent In Situ Bleomycin Toxification,” Small 17, no. 47 (2021): e2103919. 34623753 10.1002/smll.202103919

[68] G. Liao , F. He , Q. Li , et al., “Emerging Graphitic Carbon Nitride‐Based Materials for Biomedical Applications,” Progress in Materials Science 112 (2020): 100666.

[69] J. Wang and S. Wang , “A Critical Review on Graphitic Carbon Nitride (g‐C3N4)‐Based Materials: Preparation, Modification and Environmental Application,” Coordination Chemistry Reviews 453 (2022): 214338.

[70] J. Xia , C. Hu , Y. Ji , et al., “Copper‐Loaded Nanoheterojunction Enables Superb Orthotopic Osteosarcoma Therapy via Oxidative Stress and Cell Cuproptosis,” ACS Nano 17, no. 21 (2023): 21134–21152. 37902237 10.1021/acsnano.3c04903

[71] Y. T. Zhong , Y. Cen , L. Xu , S. Y. Li , and H. Cheng , “Recent Progress in Carrier‐Free Nanomedicine for Tumor Phototherapy,” Advanced Healthcare Materials 12, no. 4 (2023): e2202307. 36349844 10.1002/adhm.202202307

[72] J. Zheng , H. Ge , M. Guo , et al., “Photoinduced Cuproptosis With Tumor‐Specific for Metastasis‐Inhibited Cancer Therapy,” Small 20, no. 10 (2024): e2304407. 37880907 10.1002/smll.202304407

[73] T. T. Dongsar , T. S. Dongsar , N. Gupta , W. H. Almalki , A. Sahebkar , and P. Kesharwani , “Emerging Potential of 5‐Fluorouracil‐Loaded Chitosan Nanoparticles in Cancer Therapy,” Journal of Drug Delivery Science and Technology 82 (2023): 104371.

[74] J. Ding and Y. Guo , “Recent Advances in Chitosan and Its Derivatives in Cancer Treatment,” Frontiers in Pharmacology 13 (2022): 888740. 35694245 10.3389/fphar.2022.888740 PMC9178414

[75] R. Zheng , Y. Cheng , F. Qi , et al., “Biodegradable Copper‐Based Nanoparticles Augmented Chemodynamic Therapy Through Deep Penetration and Suppressing Antioxidant Activity in Tumors,” Advanced Healthcare Materials 10, no. 14 (2021): e2100412. 34075731 10.1002/adhm.202100412

[76] Q. Fu , L. Yu , Y. Wang , P. Li , and J. Song , “Biomarker‐Responsive Nanosystems for Chronic Disease Theranostics,” Advanced Functional Materials 33 (2022): 2206300.

[77] L. Chen , J. Min , and F. Wang , “Copper Homeostasis and Cuproptosis in Health and Disease,” Signal Transduction and Targeted Therapy 7, no. 1 (2022): 378. 36414625 10.1038/s41392-022-01229-y PMC9681860

[78] D. A. Da Silva , A. De Luca , R. Squitti , et al., “Copper in Tumors and the Use of Copper‐Based Compounds in Cancer Treatment,” Journal of Inorganic Biochemistry 226 (2022): 111634. 34740035 10.1016/j.jinorgbio.2021.111634

[79] M. T. Kuo , S. Fu , N. Savaraj , and H. H. W. Chen , “Role of the Human High‐Affinity Copper Transporter in Copper Homeostasis Regulation and Cisplatin Sensitivity in Cancer Chemotherapy,” Cancer Research 72, no. 18 (2012): 4616–4621. 22962276 10.1158/0008-5472.CAN-12-0888 PMC3445735

[80] Y. Zhang , J. Zhu , H. Sun , and J. Li , “Modulation of Tumor Hypoxia and Redox Microenvironment Using Nanomedicines for Enhanced Cancer Photodynamic Therapy,” Applied Materials Today 29 (2022): 101687.

[81] F. Sun , Y. Chen , K. W. K. Lam , et al., “Glutathione‐Responsive Aggregation‐Induced Emission Photosensitizers for Enhanced Photodynamic Therapy of Lung Cancer,” Small 20, no. 40 (2024): e2401334. 38804884 10.1002/smll.202401334

[82] J. Zhuang , G. Qi , Y. Feng , et al., “Thymoquinone as an Electron Transfer Mediator to Convert Type II Photosensitizers to Type I Photosensitizers,” Nature Communications 15, no. 1 (2024): 4943. 10.1038/s41467-024-49311-z PMC11164902 38858372

[83] X. Meng , Y. Han , S. Wang , et al., “Near‐Infrared Photosensitizers Adaptive to Tumor Hypoxic Microenvironment for Synergistic Photothermal‐Photodynamic and Immunotherapy,” Nano Today 53 (2023): 102030.

