# Correction to “Nanoparticle‐Mediated Cuproptosis and Photodynamic Synergistic Strategy: A Novel Horizon for Cancer Therapy”

**DOI:** 10.1002/cam4.71022

**Published:** 2025-07-23

Zhang, J., Zhang, A., Guo, Y., Miao, G., Liang, S., Wang, J. and Wang, J. (2025), Nanoparticle‐Mediated Cuproptosis and Photodynamic Synergistic Strategy: A Novel Horizon for Cancer Therapy. Cancer Med, 14: e70599. https://doi.org/10.1002/cam4.70599.

In the corresponding author section, the positions of the two corresponding authors are reversed. Wang Junhong is designated as the first corresponding author, while Wang Jie is listed as the second corresponding author. This article is free from any conflicts of interest, and all co‐authors have consented to the modification of the corresponding author sequence.

We apologize for this error.

